# Infection-driven gut dysbiosis and epigenetic programming of microglia: toward a systems level framework linking microbial metabolites, neuroinflammation, synaptic dysfunction, and probiotic modulation

**DOI:** 10.3389/fcimb.2026.1815265

**Published:** 2026-07-24

**Authors:** Delin Han, Chunyu Yang

**Affiliations:** School of Bioengineering, Qilu University of Technology (Shandong Academy of Sciences), Jinan, Shandong, China

**Keywords:** epigenetic reprogramming, infection-driven dysbiosis, neuroinflammation, probiotic, probiotic modulation

## Abstract

Neurodegenerative disorders such as Alzheimer’s and Parkinson’s diseases are increasingly viewed as conditions influenced by systemic immune and metabolic disturbances beyond the central nervous system (CNS). Emerging evidence suggests that infection-driven intestinal dysbiosis may function as an upstream contributor to systemic inflammation through disruption of gut barrier integrity. Increased permeability can facilitate the translocation of microbial components and metabolites into circulation, potentially influencing host immune programming via epigenetic mechanisms. Among CNS immune cells, microglia appear particularly susceptible to such peripheral cues due to their longevity and capacity for stimulus-dependent transcriptional adaptation. Experimental studies indicate that transient systemic immune challenges may induce persistent chromatin-level alterations within microglial regulatory regions, thereby reshaping their responsiveness to subsequent inflammatory stimuli or age-related stressors, including oxidative stress, chronic low-grade inflammation (inflammaging), and age-associated decline in cellular homeostatic and repair mechanisms. This phenomenon, often described as innate immune memory, may contribute to sustained neuroinflammatory activity and impaired synaptic function across the lifespan. Microbiota-derived metabolites, including short-chain fatty acids and tryptophan catabolites, have been implicated in modulating host transcriptional pathways through histone deacetylase inhibition and receptor-mediated signaling.

## 1 Introduction

Neurodegenerative disorders (NDDs) represent one of the most pressing biomedical challenges of the twenty-first century, not only because of their rising prevalence but also due to their striking heterogeneity in onset, progression, and clinical expression. Individuals exposed to seemingly similar genetic risks and environmental influences often follow markedly different neurocognitive trajectories. This variability suggests that disease vulnerability may be shaped by modulatory systems operating beyond classical protein aggregation paradigms (Fernández‐Ruiz, 2019; Martinelli et al., 2019). Increasingly, attention has shifted toward systemic and environmental contributors that influence how the central nervous system (CNS) responds to stress across the lifespan. Among these, peripheral immune perturbations and intestinal ecosystem dynamics have emerged as important determinants of neuroinflammatory tone (Cannon and Greenamyre, 2011). The gut–brain axis has reshaped contemporary understanding of brain–body communication. The brain is no longer viewed as an isolated organ. Instead, it operates within a bidirectional network involving immune mediators, metabolic intermediates, microbial metabolites, and neural circuits. The intestinal microbiota represents a dynamic ecological system that can influence host physiology far beyond digestion (Rutsch et al., 2020). Early research in this field mainly focused on compositional differences in microbial taxa associated with neurological disease. However, a more functional perspective is now emerging. This perspective emphasizes regulatory and metabolic interactions rather than taxonomy alone (Vanuytsel et al., 2023). In parallel with advances in microbiome research, the concept of immune programming has gained importance. Peripheral inflammatory events, whether triggered by infection, barrier dysfunction, or systemic immune activation, may not only generate transient cytokine responses. Instead, evidence suggests that these events can recalibrate immune responsiveness even after the initial stimulus has resolved. This recalibration may occur through mechanisms that alter transcriptional accessibility, reshape signaling thresholds, or modify the balance between pro- and anti-inflammatory pathways (Ilott et al., 2016). Within this framework, the key question is no longer whether peripheral inflammation affects the brain. Instead, it is whether repeated or unresolved immune perturbations can establish durable neuroimmune set-points that influence vulnerability to later-life pathology.

One potential mediator of these long-term effects is epigenetic regulation. Chromatin architecture provides a biologically plausible mechanism through which environmental signals can be encoded without changing DNA sequence. Modifications in histone acetylation, methylation, and enhancer accessibility allow cells to adjust gene expression in response to external stimuli (Gräff et al., 2011; Mazzio and Soliman, 2012). Importantly, these regulatory layers are dynamic but also capable of persistence. This allows prior exposures to shape future cellular responses. In immune cells, including tissue-resident macrophages, chromatin remodeling has been linked to innate immune memory. This memory can bias subsequent inflammatory responses. Whether similar mechanisms operate in infection-associated microbial disturbances and neurodegenerative susceptibility remains an open question (Schultze, 2017; Zhang and Cao, 2021). Microglia are long-lived resident immune cells of the CNS. They continuously survey the brain microenvironment. They also integrate signals from systemic circulation, metabolic status, and neuronal activity. Experimental studies show that peripheral immune challenges can alter microglial inflammatory thresholds and cytokine responses later in life (Bilbo et al., 2006; Godbout et al., 2008). These findings suggest that microglial priming may represent a cumulative record of peripheral immune history. Microglia may therefore not act only as passive responders to neuronal pathology. Instead, they may actively shape neurodegenerative trajectories through state-dependent inflammatory regulation.

At the level of the intestinal ecosystem, systemic infection has been associated with reproducible changes in microbial balance and barrier integrity. These changes can increase exposure to microbial-derived products (Yeoh et al., 2021). Such peripheral disturbances suggest that infection-driven dysbiosis may influence regulatory networks beyond the gut. Moreover, microbial metabolites, including short-chain fatty acids (SCFAs) and tryptophan-derived compounds, can interact with chromatin-modifying enzymes and transcriptional regulators under defined experimental conditions (Chang et al., 2014; Tan et al., 2022). Most mechanistic evidence comes from peripheral tissues. However, these findings suggest that microbiota-derived signals may intersect with host epigenetic systems that regulate immune responsiveness across tissues and time. Preclinical models of neurodegeneration have also investigated whether targeted modulation of microbial communities can influence synaptic resilience and inflammatory signaling (Asl et al., 2019; Li et al., 2022). Although these findings are context-dependent and require careful interpretation, they suggest that microbiota–immune–brain interactions are not merely correlative. Instead, they may represent modifiable axes within complex disease networks. Importantly, such interventions can affect not only inflammatory mediators but also molecular pathways related to synaptic stability and cellular stress responses. This highlights the interconnected nature of immune and neuronal regulation (Fung et al., 2017; Cryan et al., 2019). Despite these advances, a fully integrated framework linking infection-associated dysbiosis, epigenetically mediated immune programming, and microglial responsiveness across the lifespan has not yet been systematically established. Most existing studies examine individual components, such as microbiome alterations, epigenetic regulation, synaptic dysfunction, or neuroinflammation, in isolation. The central premise of this review is that these domains may converge within a shared regulatory architecture. In this model, peripheral immune perturbations—particularly those associated with infection and barrier dysfunction—interact with chromatin-level mechanisms. Together, these interactions shape long-term neuroimmune trajectories (Miro-Blanch and Yanes, 2019; Morais et al., 2021). Accordingly, this review synthesizes evidence from experimental and translational research to explore how infection-driven intestinal disturbances may interact with host epigenetic machinery and influence microglial programming (i.e., long-term functional and transcriptional adaptation of microglia in response to environmental or immune stimuli), synaptic vulnerability (i.e., increased susceptibility of synapses to dysfunction or loss under inflammatory or stress conditions), and neuroinflammatory outcomes. By integrating insights from microbial ecology, immune biology, and chromatin regulation, the aim is to outline a systems-level perspective that extends beyond descriptive associations toward mechanistic plausibility. Although causal relationships remain to be fully established, clarifying how peripheral environmental exposures shape central immune states may represent an important step toward identifying modifiable determinants of neurodegenerative susceptibility. Importantly, previous research has often examined microbiome alterations in neurodegeneration, epigenetic regulation of immune responses, and microglial priming largely in isolation. The present review therefore advances a conceptual synthesis in which infection-associated intestinal dysbiosis and epithelial barrier perturbation are viewed not merely as correlates of systemic inflammation, but as potential upstream modulators of chromatin-level immune programming with implications for long-term neuroinflammatory set-points. This integrative framework aims to clarify how peripheral immune perturbations may contribute to variability in neurodegenerative vulnerability while maintaining a cautious distinction between biologically plausible mechanisms and established causality.

## 2 Microbiota–gut–brain communication in neurological homeostasis: neural signaling, microbial metabolites, and dysbiosis-driven neurodegeneration

The gut–brain axis is a complex, bidirectional communication system that connects the gastrointestinal tract and the CNS through neural, hormonal, metabolic, and immune pathways. It plays a critical role in maintaining neurological homeostasis and is increasingly recognized for its involvement in major NDDs, including Alzheimer’s disease (AD) and Parkinson’s disease (PD) (Caradonna et al., 2024). Key players in this interaction include the vagus nerve, the hypothalamic–pituitary–adrenal axis (HPA), the immune system, and microbial-derived metabolites such as SCFAs, neurotransmitters, and lipopolysaccharide (LPS) (Forsythe et al., 2014; Carlessi et al., 2021; Rusch et al., 2023). The vagus nerve, the tenth cranial nerve, is a central component of the gut–brain axis and serves as a primary channel for neural signals from the gut to the brain. It connects the enteric nervous system with the brainstem and can transmit information in both directions (Chuyue et al., 2020). Evidence suggests that alpha-synuclein, a misfolded protein associated with PD, may propagate along this route from the gut to the brain. Enteroendocrine cells (EECs), which possess neuron-like properties and express alpha-synuclein, form direct synaptic connections with enteric neurons. These neuropod-containing cells are capable of sensing dietary and microbial signals and relaying them via synaptic-like processes, providing a direct neuroepithelial interface between the gut lumen and the nervous system (Chandra et al., 2017). Microbial metabolites further modulate this communication network. SCFAs, particularly butyrate, propionate, and acetate, are produced by bacterial fermentation of dietary fibers (Kaur et al., 2025).

These molecules influence various aspects of host physiology, including immune regulation, gut barrier integrity, and brain function. SCFAs can cross the blood–brain barrier, where they interact with microglia, modulating neuroinflammatory responses and promoting neuronal homeostasis. They also influence the differentiation of regulatory T cells and stimulate the release of gut hormones via EECs, reinforcing gut–brain communication (Haase et al., 2020; Filippone, 2021). Conversely, gut dysbiosis—an imbalance in microbial composition—has been implicated in the pathophysiology of both AD and PD. In PD, a significant reduction in SCFA-producing bacteria such as Faecalibacterium and Prevotella is often reported, accompanied by an increase in pro-inflammatory taxa like Enterobacteriaceae. These alterations are associated with increased intestinal permeability, also known as “leaky gut,” allowing LPS and other microbial products to enter the systemic circulation. This triggers widespread inflammation, disrupts the blood–brain barrier (BBB), and promotes alpha-synuclein aggregation in the brain, contributing to dopaminergic neuron degeneration (Petrov et al., 2017; Drago et al., 2019; Pietrucci et al., 2019). Similar patterns of dysbiosis have been observed in AD. Patients with AD show lower levels of beneficial bacteria and higher proportions of pro-inflammatory species. This microbial imbalance correlates with increased levels of LPS and bacterial amyloids in the gut, which may activate immune responses and promote amyloid-beta (Aβ) aggregation in the brain. Certain gut bacteria, including *Escherichia coli* and Salmonella, produce curli-type amyloid fibers capable of inducing cross-seeding of misfolded proteins such as Aβ and tau. These bacterial amyloids can be internalized by enteric neurons and potentially travel to the CNS, promoting the formation of amyloid plaques and neurofibrillary tangles (Aboytes Flores, 2023; Ali et al., 2023). Studies in animal models support the idea that gut microbiota influence brain pathology. Germ-free transgenic mice show reduced Aβ deposition, which increases upon colonization with microbiota from AD mice. Similarly, rodents exposed to curli-producing *E. coli* strains exhibit elevated brain alpha-synuclein levels and increased neuroinflammation compared to controls. These findings suggest that microbial factors contribute to protein aggregation pathways central to NDDs (Levi et al., 2003; Harach et al., 2017; Sampson et al., 2020). Beyond neural signaling, gut microbes also impact neuroendocrine functions. Bacterial metabolites influence the HPA axis, which regulates stress responses and hormonal secretion. Microbes also produce neuroactive compounds such as serotonin, γ-aminobutyric acid (GABA), and dopamine, which can affect behavior, memory, and synaptic plasticity (Farzi et al., 2018; Xu et al., 2024).

## 3 Epigenetic regulation in neurodegeneration: DNA methylation, histone modifications, and miRNA networks in AD and PD

Epigenetic mechanisms play a crucial role in regulating gene expression without altering the underlying DNA sequence. These mechanisms include DNA methylation, histone modifications, and non-coding RNAs, all of which are central to brain development, cognitive function, and the pathogenesis of NDDs (Jakovcevski and Akbarian, 2012). During aging, disruptions in these regulatory systems can contribute to conditions such as AD and PD (Mastroeni et al., 2011). DNA methylation involves the addition of a methyl group to the cytosine base in CpG dinucleotides, a process governed by DNA methyltransferases. This modification can silence genes or reduce their expression and is essential for maintaining cellular identity and genomic stability (Xu et al., 2019; He et al., 2021). In the nervous system, DNA methylation is highly dynamic and responsive to environmental stimuli. Neuronal activity has been shown to induce methylation changes, influencing synaptic plasticity and memory consolidation. For example, alterations in methylation patterns have been observed in genes associated with long-term potentiation, a process critical for learning (Yu et al., 2011; Halder et al., 2016). Age-associated epigenetic drift and methylation instability have been linked to progressive neuronal vulnerability and neurodegenerative susceptibility (Berni Canani et al., 2012; Stein and Riber, 2023). Histone modifications—such as methylation, acetylation, phosphorylation, and ubiquitination—alter chromatin structure and gene accessibility. These modifications are catalyzed by specific enzymes (e.g., histone acetyltransferases and deacetylases) and regulate transcriptional activity in neurons. Studies have demonstrated that learning-related experiences trigger specific histone changes in hippocampal neurons, including increased acetylation at histone H3 and H4 sites. Chromatin immunoprecipitation sequencing studies in microglia have revealed enrichment of active histone marks such as H3K27ac and H3K9ac at promoters of inflammatory genes during neuroinflammatory activation, suggesting epigenetic priming of immune responses.

Assay for Transposase-Accessible Chromatin sequencing further demonstrates increased chromatin accessibility in LPS-stimulated microglia, particularly in enhancer regions associated with NF-κB signaling, highlighting dynamic epigenetic remodeling during inflammation. Non-coding RNAs, particularly miRNAs, further expand epigenetic regulatory complexity. These small RNA molecules (18–25 nucleotides) bind to complementary sequences on target mRNAs, resulting in translational repression or degradation. Some miRNAs are themselves regulated by DNA methylation and histone modifications, forming feedback loops with epigenetic machinery. In turn, miRNAs regulate key epigenetic enzymes, establishing tightly controlled gene regulatory networks (Hombach and Kretz, 2016; Bure et al., 2022). Dysregulation of miRNAs has been widely implicated in neurodegeneration. In AD, altered levels of miR-34a, miR-124, and miR-9 have been linked to Aβ accumulation, tau phosphorylation, and neuroinflammation (Du et al., 2017; Lee et al., 2021). Similarly, in PD, miR-155, let-7a, and miR-222 are associated with dopaminergic neuronal loss and inflammation (Vallelunga et al., 2014; Elangovan et al., 2023). Emerging evidence highlights a strong interaction between the gut microbiota and host epigenome in aging and neurodegeneration (Xu et al., 2019). Gut microbes produce metabolites—especially SCFAs such as butyrate, acetate, and propionate—that cross the BBB and regulate epigenetic mechanisms in neural tissue. Butyrate acts as a histone deacetylase (HDAC) inhibitor, promoting histone acetylation and enhancing transcription of anti-inflammatory and neuroprotective genes (Berni Canani et al., 2012; Stein and Riber, 2023). Moreover, LPS derived from Gram-negative bacteria activates innate immune signaling via Toll-Like Receptor 4 (TLR4)/Nuclear Factor kappa B (NF-κB) pathways and induces epigenetic remodeling. LPS exposure has been associated with alterations in DNA methylation and histone modification patterns, leading to persistent inflammatory gene activation in neural and immune cells (Zhang and Ghosh, 2000a; Yang et al., 2021; Liu et al., 2022). Importantly, these microbial–epigenetic interactions extend to non-coding RNA regulation. LPS-induced signaling has been shown to upregulate pro-inflammatory miRNAs such as miR-34a, miR-146a, and miR-155, which target neuroprotective genes including NF-L, thereby contributing to axonal injury and synaptic dysfunction in AD (Liu et al., 2022). Furthermore, LPS-triggered NF-κB activation promotes transcriptional reprogramming of immune cells through miRNA-mediated feedback loops, reinforcing chronic neuroinflammatory states (Zhang and Ghosh, 2000b). In parallel, circulating exosomal miRNAs have been shown to correlate with gut microbial composition and cognitive decline in AD, supporting a microbiota–gut–brain axis in which microbial dysbiosis is linked to systemic miRNA signatures involved in disease progression (Gui et al., 2015; Lin et al., 2025). These miRNAs regulate genes involved in synaptic transmission, neurotrophin signaling, and cellular stress responses, suggesting a functional bridge between microbiota and neuronal gene regulation (Issler and Chen, 2015). Similarly, altered expression of inflammation-related microRNAs, including miR-155 and miR-146a, has been associated with dopaminergic neuronal loss and activation of neuroinflammatory signaling pathways in PD models, further supporting a microbiota–immune–miRNA regulatory axis in neurodegeneration (Gui et al., 2015; Yang et al., 2021).

## 4 Microbiota-derived SCFAs as epigenetic modulators of neuroinflammation

Recent discoveries linking the gut microbiome to neurological health have highlighted the role of microbial metabolites, particularly SCFAs, in gut–brain communication and neuroimmune regulation. SCFAs—mainly acetate, propionate, and butyrate—are produced in the colon through bacterial fermentation of dietary fiber and serve as key biochemical mediators connecting intestinal dysbiosis with host physiological and epigenetic responses. Importantly, in the context of infection-associated dysbiosis, SCFA production is often reduced, leading to a shift from anti-inflammatory to pro-inflammatory signaling states that may contribute to systemic immune activation and neuroinflammation. Among their many functions, SCFAs influence gene expression in the CNS by modulating epigenetic mechanisms, including HDAC inhibition and regulation of DNA methylation (Dong and Cui, 2022; Song et al., 2022). These effects collectively contribute to the regulation of immune responses, neuronal plasticity, and cognitive function, particularly during aging and NDDs. Butyrate is the most extensively studied SCFA due to its potent HDAC inhibitory activity. HDACs remove acetyl groups from histone proteins, resulting in chromatin condensation and transcriptional repression (Khan and Jena, 2015). By inhibiting HDAC activity, butyrate promotes chromatin relaxation and facilitates transcription of genes involved in anti-inflammatory and neuroprotective pathways (Patnala et al., 2017). Mechanistically, this epigenetic modulation is particularly relevant in microglia, where chromatin accessibility determines the magnitude of inflammatory gene expression in response to peripheral immune signals.

**Figure 1:**
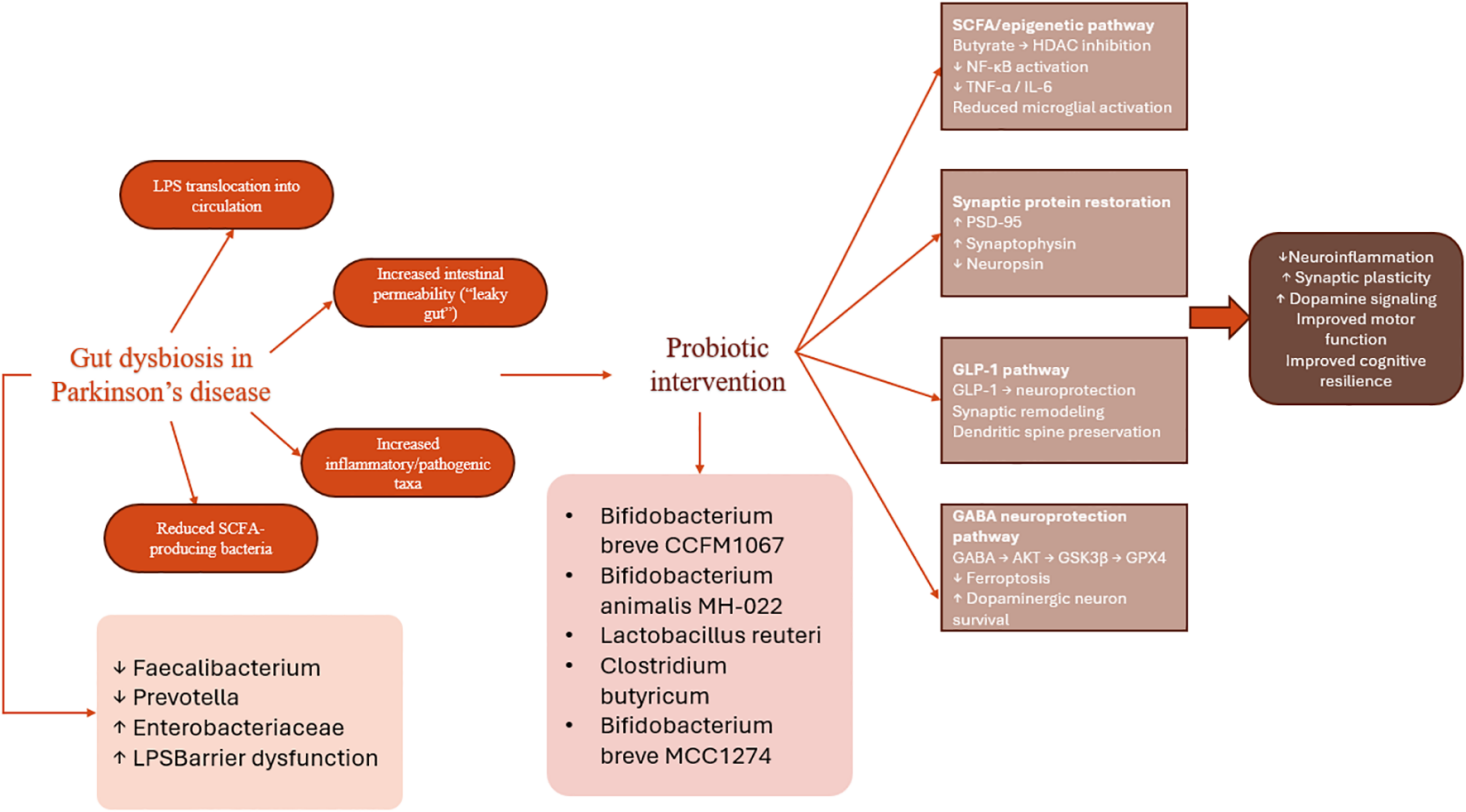
Probiotic-mediated regulation of synaptic plasticity and neuroprotection in Parkinson’s disease through the gut–brain axis. Parkinson’s disease (PD)-associated gut dysbiosis is characterized by reduced levels of beneficial short-chain fatty acid (SCFA)-producing bacteria and increased abundance of pro-inflammatory taxa, leading to intestinal barrier dysfunction and systemic translocation of microbial products such as lipopolysaccharide (LPS). These alterations promote systemic inflammation, microglial activation, and dopaminergic neurodegeneration. Probiotic interventions including *Bifidobacterium breve* CCFM1067, *Bifidobacterium animalis* MH-022, *Lactobacillus reuteri*, *Clostridium butyricum*, and *Bifidobacterium breve* MCC1274 modulate gut microbial composition and restore metabolic homeostasis through the production of neuroactive metabolites such as butyrate, acetate, and γ-aminobutyric acid (GABA). SCFAs, particularly butyrate, inhibit histone deacetylases (HDACs), suppress NF-κB-mediated inflammatory signaling, and reduce the production of pro-inflammatory cytokines including TNF-α and IL-6. GABA-mediated activation of the AKT/GSK3β/GPX4 pathway attenuates ferroptosis and enhances neuronal antioxidant defense, while GLP-1 signaling contributes to synaptic remodeling and neuronal survival. These probiotic-mediated mechanisms collectively preserve dopaminergic neurons, restore synaptic proteins such as PSD-95 and synaptophysin, improve dendritic spine integrity, and reduce neuroinflammation. Overall, modulation of the gut–brain axis by probiotics may represent a promising therapeutic strategy for maintaining synaptic plasticity and improving motor and cognitive outcomes in PD.

In primary microglial cultures, butyrate has been shown to suppress NF-κB activation, a central transcription factor in inflammatory signaling. This results in reduced expression of pro-inflammatory cytokines such as iInterleukin-6 (IL-6) and tumor necrosis factor-alpha (TNF-α), demonstrating a direct link between SCFA-mediated epigenetic regulation and neuroinflammatory suppression (Sun et al., 2025) (**Figure 1**). Experimental studies using LPS to stimulate inflammation in microglial cells and hippocampal slice cultures have demonstrated the anti-inflammatory effects of sodium butyrate. Pretreatment with butyrate attenuated the production of nitric oxide and inflammatory cytokines in response to LPS (Huuskonen et al., 2004). This suggests that SCFAs not only regulate baseline epigenetic states but also reshape stimulus-dependent inflammatory responsiveness, consistent with the concept of epigenetically mediated immune priming. Interestingly, in immortalized microglial cell lines, butyrate and other SCFAs such as propionate and valerate have been reported to enhance inflammatory responses under certain conditions (Wang et al., 2017; Xiao et al., 2018). SCFAs also play a significant role in brain aging and neuroimmune regulation. With aging, systemic inflammation increases and is accompanied by microglial overactivation and increased vulnerability to immune challenges. In aged animal models, dietary fiber supplementation (e.g., inulin) increases colonic SCFA production, particularly butyrate, and is associated with reduced peripheral inflammation and a shift in microglial gene expression toward an anti-inflammatory phenotype (Muthyala et al., 2022; Hutchinson et al., 2023). These findings support a mechanistic continuum in which dietary modulation of gut microbiota influences SCFA availability, which in turn regulates microglial transcriptional programs and neuroinflammatory tone.

In addition to butyrate, acetate also exerts important effects on brain function. Acetate crosses the BBB and is taken up by brain regions such as the hypothalamus, where it contributes to energy metabolism and regulation of neuropeptides involved in appetite control. It also influences acetyl-CoA availability, thereby indirectly modulating histone acetylation status in neural and glial cells (Deelchand et al., 2009; Hasegawa et al., 2012). Thus, both butyrate and acetate function as metabolic–epigenetic coupling molecules linking gut microbial activity to CNS transcriptional regulation. Beyond metabolic regulation, SCFAs also influence BBB integrity. SCFA supplementation has been shown to attenuate stress-induced behavioral deficits and restore barrier integrity without major changes in microbial composition or receptor expression (Li et al., 2016; Yan et al., 2022). This suggests that SCFAs act primarily through intracellular signaling and epigenetic regulation rather than receptor abundance changes, reinforcing their role as transcriptional modulators rather than classical ligands. Further supporting their neuroprotective role, sodium butyrate has demonstrated efficacy in cerebral ischemia-reperfusion models, improving neurological outcomes and preserving brain structure following injury (Sun et al., 2015; Mirzaei et al., 2022). These effects are associated with reduced oxidative stress, decreased pro-inflammatory cytokine production (IL-1β, IL-8, TNF-α), and reduced neuronal apoptosis. At the molecular level, butyrate modulates apoptosis-related proteins by decreasing Bax expression and increasing Bcl-2, phosphorylated Akt, and brain-derived neurotrophic factor (BDNF), suggesting activation of survival pathways such as PI3K/Akt signaling. Collectively, these effects indicate that SCFAs exert multimodal neuroprotection through integrated epigenetic, anti-inflammatory, antioxidant, and pro-survival mechanisms. Moreover, there is increasing recognition that SCFAs can influence behavior and cognition, possibly by altering gene expression patterns through their epigenetic actions (Stilling et al., 2016). In stress models, SCFA supplementation partially reversed deficits in reward-seeking behavior and reduced intestinal permeability without significantly affecting body weight, fecal SCFA content, or expression of SCFA receptors (FFAR2 and FFAR3) (van de Wouw et al., 2018). At a systems level, emerging evidence suggests that SCFAs act as epigenetic signaling intermediates that translate environmental inputs such as diet, stress, and microbiota composition into chromatin-level regulation of gene expression in the brain (He et al., 2020; Zhang et al., 2021; Nshanian et al., 2025). In conclusion, SCFAs—particularly butyrate and acetate—function as central epigenetic mediators linking gut microbial dysbiosis to neuroinflammation. Through HDAC inhibition, NF-κB modulation, and regulation of microglial transcriptional states, they provide a mechanistic bridge between peripheral metabolic signals and CNS immune regulation. Targeting SCFA pathways may therefore represent a promising strategy for modulating neuroinflammatory and neurodegenerative processes.

## 5 Probiotic regulation of synaptic plasticity in PD: mechanisms at the gut–brain interface

PD, traditionally characterized by motor dysfunction and dopaminergic neuronal loss, has increasingly been associated with alterations in gut microbiota composition, immune activation, and synaptic dysregulation within central neural circuits (Park et al., 2023). Within a systems-level framework, PD can be viewed not only as NDDs but also as a disorder of gut–immune–brain communication, where microbial imbalance and metabolite signaling contribute to synaptic and neuronal dysfunction. Emerging evidence suggests that probiotics may exert beneficial effects not only through anti-inflammatory and antioxidant mechanisms but also by modulating synaptic plasticity, neuronal connectivity, and epigenetic regulation of gene expression. Importantly, probiotic effects are increasingly understood as strain-specific and mechanistically heterogeneous, involving microbial restoration, metabolite production (e.g., SCFAs, GABA), and modulation of host immune–epigenetic signaling pathways. In this context, several probiotic strains—including *Bifidobacterium breve*, *Bifidobacterium animalis*, *Lactobacillus reuteri*, and *Clostridium butyricum*—have shown beneficial effects in experimental PD models, particularly through converging immune, metabolic, and synaptic pathways (**Table 1**; **Figure 1**).

**Table 1:** Synaptic plasticity, probiotic intervention, and Parkinson’s disease: An integrated perspective.

Probiotic strain	Animal model/PD induction	Duration of treatment	Mechanism explored	Effect on synaptic plasticity	Gut microbiota modulation	SCFA levels	Key outcomes	Ref
B. breve CCFM1067	MPTP-induced mice	5 weeks	Anti-inflammatory, antioxidant, BBB and gut barrier protection	Not directly assessed	↑ Bifidobacterium, Akkermansia; ↓ Escherichia-Shigella	↑ Acetate, Butyrate	Protected dopaminergic neurons; reduced glial activation and inflammation	(Li et al., 2022)
B. animalis subsp. lactis MH-022	6-OHDA-induced rats	Not specified	Anti-inflammatory, mitochondrial function, antioxidant activity	Not directly assessed	Promoted beneficial taxa	↑ SCFA production	Improved motor function, reduced inflammation, protected neurons	(Tsao et al., 2024)
L. reuteri (via GABA)	MPTP-induced mice	Not specified	Ferroptosis inhibition via AKT-GSK3β-GPX4 axis	Not directly assessed	Not specified	Not mentioned	Alleviated PD symptoms via GABA signaling and ferroptosis inhibition	(Dong et al., 2025)
Clostridium butyricum	MPTP-induced mice	4 weeks	Gut microbiota–GLP-1 signaling pathway	Improved synaptic dysfunction	Restored microbiota composition	Not directly mentioned	Ameliorated motor deficits, neuron loss, and microglia activation	(Sun et al., 2021)
B. breve A1 (MCC1274)	MPTP-induced mice	4 days	Neuropsin modulation	Restored postsynaptic protein and spine density	Not specified	Not mentioned	Improved hippocampal plasticity and memory extinction	(Ishii et al., 2021)

MPTP, 1-methyl-4-phenyl-1,2,3,6-tetrahydropyridine; BBB, blood–brain barrier; 6-OHDA, 6-hydroxydopamine; AKT–GSK3β–GPX4 axis, protein kinase B–glycogen synthase kinase-3 beta–glutathione peroxidase 4 signaling pathway; GABA, gamma-aminobutyric acid.

### 5.1 Gut microbial restoration and SCFA-mediated epigenetic–synaptic coupling

Across multiple experimental models, probiotics consistently restore gut microbial balance by increasing beneficial taxa such as Bifidobacterium and Akkermansia while reducing pro-inflammatory bacteria such as *Escherichia–Shigella*. This microbial reshaping is consistently associated with increased production of SCFAs, particularly acetate and butyrate, which function as key epigenetic regulators through HDAC inhibition and modulation of inflammatory gene transcription. In 1-methyl-4-phenyl-1,2,3,6-tetrahydropyridine and 6-hydroxydopamine-6 models, this SCFA increase correlates with reduced dopaminergic neurodegeneration, improved mitochondrial function, and suppression of neuroinflammatory signaling (Li et al., 2022; Tsao et al., 2024). Mechanistically, these findings suggest that probiotic-induced neuroprotection is mediated not only by anti-inflammatory effects but also by SCFA-driven epigenetic reprogramming of neuronal and glial cells, leading to a more anti-inflammatory transcriptional state.

### 5.2 Metabolite-specific neuroprotection and synaptic stabilization

Beyond SCFAs, microbial metabolites such as GABA have been identified as direct neuromodulators. *Lactobacillus reuteri*–derived GABA has been shown to suppress ferroptosis through activation of the AKT–GSK3β–GPX4 signaling pathway, thereby protecting dopaminergic neurons and preserving synaptic integrity (Dong et al., 2025). This highlights a second mechanistic axis in which probiotics influence synaptic stability through direct neurotransmitter production, linking microbial metabolism to neuronal survival pathways independent of classical immune modulation.

### 5.3 Gut–brain hormonal signaling and synaptic plasticity

Probiotics such as *Clostridium butyricum* enhance glucagon-like peptide-1 (GLP-1) signaling both in the gut and brain, promoting neuronal survival, synaptic remodeling, and motor recovery (Sun et al., 2021). GLP-1–mediated effects represent a gut–brain endocrine pathway that converges with SCFA- and immune-mediated signaling, suggesting that probiotic interventions act through multi-layered regulatory networks rather than single pathways. Additionally, normalization of SCFA receptors (GPR41/43) indicates that probiotic effects extend to receptor-level reprogramming of host metabolic sensing systems.

### 5.4 Hippocampal plasticity and synaptic protein remodeling

In hippocampus-dependent cognitive domains, *Bifidobacterium breve* A1 restores synaptic proteins such as PSD-95 and synaptophysin and normalizes neuropsin expression, while also improving dendritic spine density in CA1 neurons (Ishii et al., 2021). These effects indicate that probiotics not only modulate neurotransmitter systems but also directly regulate synaptic structural proteins and proteolytic enzymes involved in synaptic remodeling, thereby contributing to cognitive resilience in PD.

### 5.5 Integrated mechanistic synthesis

Collectively, these findings converge on a unified mechanistic framework in which probiotics modulate PD pathology through three interconnected axes:

1. Microbial restoration and immune rebalancing
2. Metabolite-mediated epigenetic regulation (SCFAs, GABA)
3. Hormonal and neurotransmitter-like signaling (GLP-1, GABA pathways)

These axes converge at the level of neuronal and glial cells, where they regulate chromatin accessibility, inflammatory gene expression, mitochondrial function, and synaptic protein stability. Thus, probiotic interventions should not be interpreted as isolated strain-specific effects but rather as systems-level modulators of the gut–microbiota–immune–epigenetic–synaptic axis in PD. This integrated perspective aligns with the proposed framework of infection-driven dysbiosis leading to epigenetic remodeling, microglial activation, and synaptic dysfunction.

## 6 Synaptic plasticity and probiotic intervention in AD: insights from experimental and clinical studies

**Figure 2:**
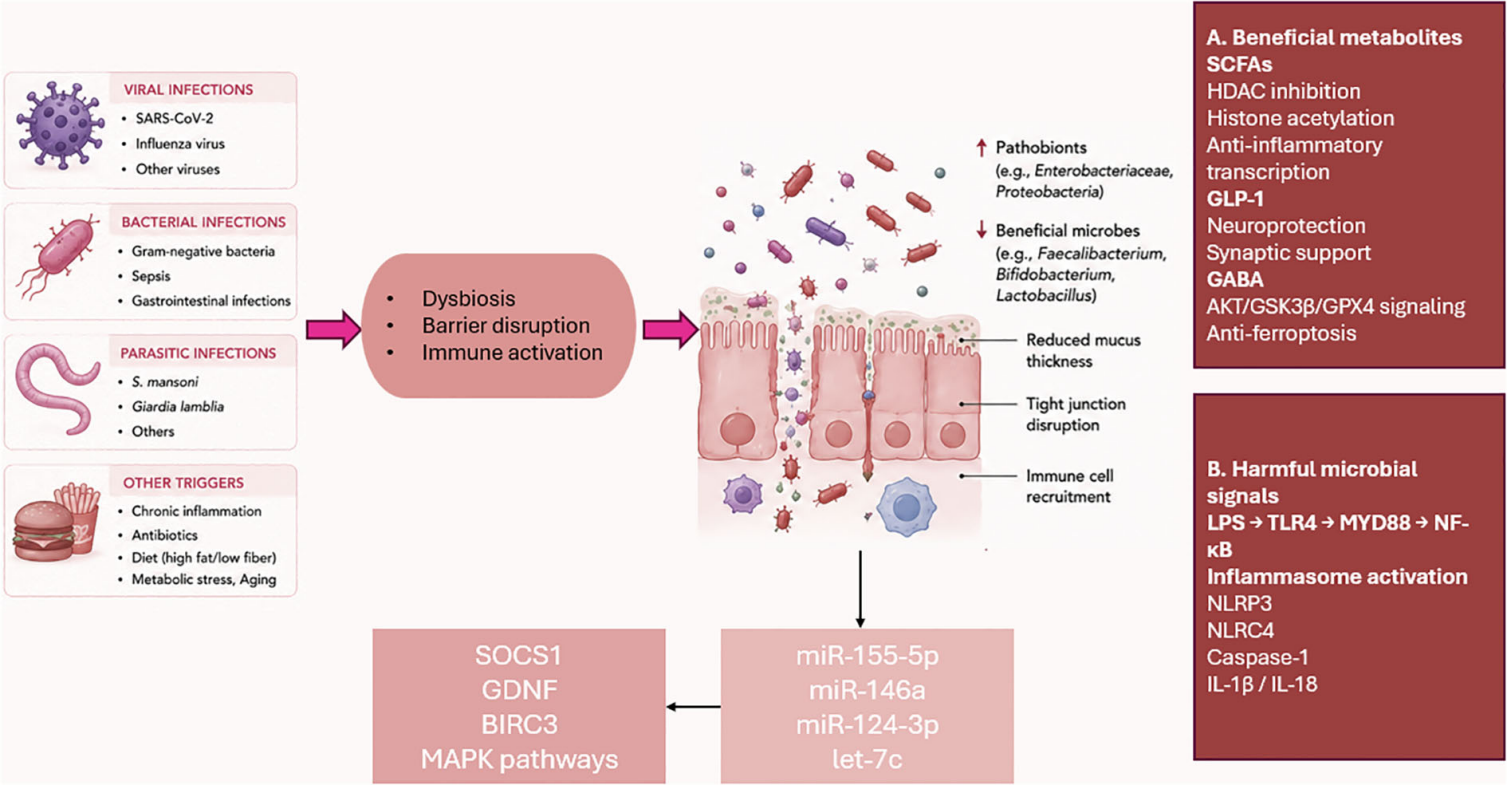
Infectious and environmental triggers initiating gut dysbiosis and intestinal barrier disruption. A variety of infectious and environmental factors contribute to the initiation of gut dysbiosis and intestinal barrier dysfunction, which represent early upstream events in gut–brain axis dysregulation. Viral infections such as SARS-CoV-2 and influenza, bacterial infections including Gram-negative pathogens and sepsis-associated microbes, and parasitic infections such as *Schistosoma mansoni* and *Giardia lamblia* can disrupt intestinal microbial homeostasis. Additional non-infectious factors—including chronic inflammation, antibiotic exposure, high-fat/low-fiber diets, metabolic stress, obesity, and aging—further exacerbate microbial imbalance and mucosal injury. Under healthy conditions, the intestinal microbiota maintains epithelial integrity through balanced microbial composition, preserved mucus thickness, and intact tight junctions. In contrast, post-infectious and inflammatory states are characterized by expansion of pathobionts (e.g., *Enterobacteriaceae* and Proteobacteria), depletion of beneficial taxa (e.g., *Faecalibacterium*, *Bifidobacterium*, and *Lactobacillus*), reduced mucus layer thickness, disruption of epithelial tight junctions, and recruitment of immune cells into the intestinal mucosa. These alterations promote increased intestinal permeability (“leaky gut”), systemic immune activation, microbial translocation, and chronic inflammation, thereby establishing a pathological foundation for downstream neuroimmune and neurodegenerative processes.

AD is characterized by progressive cognitive decline driven by synaptic loss, chronic neuroinflammation, and neuronal network dysfunction. Beyond classical amyloid and tau pathology, increasing evidence highlights AD as a systems-level disorder involving dysregulation of the gut–brain axis, in which intestinal microbiota influence brain function through immune, metabolic, and epigenetic pathways (Pasupalak et al., 2024). Importantly, this axis can be conceptualized as a continuous signaling cascade linking gut microbial imbalance to central synaptic dysfunction. Within this framework, probiotics and prebiotics are increasingly considered modulators of brain plasticity through their ability to reshape gut microbial composition and downstream host signaling rather than acting as isolated therapeutic agents (**Table 2**; **Figure 2**).

**Table 2:** Synaptic plasticity and probiotic intervention in Alzheimer’s disease: Insights from experimental and clinical studies.

Study type	Model/Subjects	Intervention	Duration	Mechanism of action	Epigenetic/Molecular targets	Neuroprotective/Inflammatory outcomes	Cognitive/Behavioral outcome	Key findings/Notes	Ref
Animal Study	β-amyloid injected rats	Probiotic mixture	Not specified	Antioxidant enhancement, synaptic plasticity restoration	TAC, MDA, LTP	Reduced oxidative stress and improved synaptic transmission	Improved spatial memory	Probiotic restored LTP and improved maze navigation	(Asl et al., 2019)
Clinical Trial	AD patients	Multi-strain probiotic	12 weeks	Increased BDNF, reduced IL-1β, increased SOD	BDNF, IL-1β, SOD	Reduced inflammation and oxidative stress	Non-significant trend toward cognitive improvement	Probiotic increased BDNF and reduced inflammatory markers	(Hsu et al., 2023)
Animal Study	APP/PS1 mice	Saccharomyces boulardii	Not specified	TLR pathway inhibition, microglia regulation	TLR, CD11b, IL-6, IL-1β	Reduced neuroinflammation, improved synaptic integrity	Improved cognitive performance	Probiotic improved microbiota and neuroinflammation	(Ye et al., 2022)
Animal Study	3×Tg-AD mice	Lactobacillus plantarum PS128	33 days	Regulated PPA, GSK3β, gliosis	GSK3β, pT231 tau	Reduced gliosis and AD pathology	Prevented cognitive dysfunction	PS128 modulates propionic acid and kinase activity	(Huang et al., 2021)
Animal Study	AppNL-G-F mice	VSL#3 probiotic	8 weeks	Increased SCFAs, especially acetate and lactate	SCFAs, c-Fos	Minimal histological change; increased c-Fos	Potential activation of neuronal pathways	Altered microbiota and SCFA levels	(Kaur et al., 2020)
Animal Study	APP/PS1 mice	Encapsulated L. plantarum	Not specified	Restored SCFA levels, gut barrier integrity	PSD-95, Aβ, tau	Reduced neuroinflammation and Aβ/tau pathology	Improved cognitive functions	LbL encapsulation improved probiotic efficacy	(Hu et al., 2024)
Animal Study	Wild-type mice	B. breve MCC1274	4 months	Akt/GSK-3β pathway modulation	GSK3β, tau, Aβ	Reduced Aβ and phosphorylated tau	Improved memory and synaptic proteins	Attenuated AD pathology in WT mice	(Ahmed Abdellatif Abdelhamid, 2023)
Animal Study	17-month-old AppNL-G-F mice	B. breve MCC1274	Not specified	Reduced JNK/ERK stress, tau phosphorylation	JNK, ERK, tau	Reduced inflammation and enhanced synaptic density	Ameliorated memory impairment	Improved neuronal activity and reduced stress	(Abdelhamid et al., 2024)
Animal Study	Aβ1–42 treated mice	B. breve CCFM1025 and WX	Not specified	Increased BDNF, FNDC5, PSD-95	BDNF, PSD-95	Improved synaptic plasticity	Improved cognition	Gut microbiota modulation improved outcomes	(Zhu et al., 2021)
Animal Study	APP/PS1 mice	FOS	6 weeks	GLP-1/GLP-1R modulation, microbiota restoration	JNK, PSD-95, synapsin I	Reduced neurodegeneration and inflammation	Ameliorated cognitive deficits	Prebiotics altered gut-brain signaling	(Sun et al., 2019)
Clinical Trial	Severe AD patients	Probiotic mixture	12 weeks	No pronounced effect in severe AD	NO, GSH, TAC, IL-6	Minimal impact on biomarkers	No cognitive improvement	AD severity affects response to probiotics	(Agahi et al., 2018)

AD, Alzheimer’s disease; BDNF, brain-derived neurotrophic factor; IL-1β, interleukin-1 beta; SOD, superoxide dismutase; APP/PS1 mice, amyloid precursor protein/presenilin-1 double-transgenic mice; TLR, Toll-like receptor; CD11b, cluster of differentiation 11b (integrin αM); IL-6, interleukin-6; GSK3β, glycogen synthase kinase-3 beta; SCFAs, short-chain fatty acids; c-Fos, cellular Fos proto-oncogene (a marker of neuronal activity); AppNL-G-F mice, knock-in Alzheimer’s disease mouse model carrying the Swedish (NL), Arctic (G), and Iberian (F) mutations in the amyloid precursor protein gene; Aβ, amyloid-beta peptide; JNK, c-Jun N-terminal kinase; ERK, extracellular signal-regulated kinase.

### 6.1 Restoration of synaptic plasticity through probiotics in animal models

Across experimental models, probiotic and prebiotic interventions consistently improve synaptic function and cognitive performance, and these effects are tightly associated with restoration of gut microbial composition and increased production of neuroactive metabolites such as SCFAs. These metabolites, particularly butyrate and acetate, are strongly implicated in epigenetic regulation through HDAC inhibition, which facilitates transcriptional activation of genes involved in synaptic maintenance and neuroprotection. Across different AD models, improvements in hippocampal synaptic plasticity, including restoration of long-term potentiation and enhancement of synaptic protein expression such as PSD-95 and synaptophysin, are commonly observed following probiotic administration (Asl et al., 2019; Hu et al., 2024). These synaptic changes occur in parallel with reductions in neuroinflammatory signaling, particularly through suppression of microglial activation and Toll-like receptor–mediated pathways, suggesting that immune modulation is a central component of probiotic action (Ye et al., 2022). In addition, several studies indicate that probiotic-induced modulation of intracellular signaling pathways such as GSK3β contributes to reduced tau phosphorylation and improved neuronal stability (Huang et al., 2021). Importantly, increases in neuronal activity markers such as c-Fos further suggest that microbial metabolites can enhance functional synaptic activation even when amyloid pathology remains relatively unchanged (Kaur et al., 2020). Taken together, these findings support a mechanistic sequence in which gut microbial modulation alters SCFA availability, which in turn reshapes epigenetic regulation of inflammatory and synaptic genes, ultimately leading to reduced microglial activation and improved synaptic function.

### 6.2 Influence of specific probiotic strains on synaptic and molecular pathways

Different probiotic strains exert overlapping yet partially distinct effects on synaptic plasticity in AD models, reflecting strain-specific modulation of gut–brain signaling pathways. Bifidobacterium breve strains, for example, improve synaptic integrity by regulating intracellular signaling cascades such as Akt/GSK3β, which are closely involved in tau phosphorylation and neuronal survival (Ahmed Abdellatif Abdelhamid, 2023; Abdelhamid et al., 2024). These molecular changes are accompanied by increased expression of synaptic proteins and reduced microglial activation, indicating simultaneous regulation of neuronal and immune components of AD pathology. Other strains such as *Lactobacillus plantarum* PS128 modulate synaptic stability through regulation of glycogen synthase kinase 3β activity, reduction of tau phosphorylation, and restoration of postsynaptic density protein 95, effects that are associated with reduced gliosis and improved metabolic balance via propionic acid–related pathways (Huang et al., 2021). Similarly, *Bifidobacterium breve* MCC1274 improves synaptic integrity and neuronal activity by suppressing stress-related signaling pathways, reducing tau hyperphosphorylation, and enhancing synaptic protein expression, thereby supporting synaptic plasticity through amyloid-independent mechanisms (Abdelhamid et al., 2024). Despite these mechanistic differences, a consistent pattern emerges in which all probiotic strains ultimately converge on the regulation of synaptic plasticity through coordinated effects on microbial metabolites, inflammatory signaling, and intracellular neuronal pathways. Importantly, these converging pathways intersect at the level of epigenetic regulation and microglial phenotype modulation, providing a unified explanation for their shared synaptic benefits.

### 6.3 Clinical studies: translational impact on human cognitive function

Clinical evidence further supports the mechanistic relevance of gut microbiota modulation in AD, although cognitive outcomes remain more variable compared to experimental models. In patients receiving multi-strain probiotic formulation containing *Bifidobacterium longum* subsp. infantis BLI-02, *Bifidobacterium breve* Bv-889, *Bifidobacterium animalis* subsp. lactis CP-9, *Bifidobacterium bifidum* VDD088, and *Lactobacillus plantarum* PL-02, increases in BDNF and antioxidant capacity, along with reductions in pro-inflammatory cytokines such as IL-1β, suggest a shift toward a more neuroprotective systemic environment (Hsu et al., 2023). These peripheral molecular changes are mechanistically consistent with central effects observed in experimental models, suggesting that systemic immune and metabolic modulation may indirectly influence brain synaptic plasticity via gut–brain signaling pathways. However, the limited cognitive improvement observed in some trials, particularly in advanced stages of AD, suggests that therapeutic efficacy is strongly dependent on disease progression and residual synaptic plasticity (Agahi et al., 2018).

## 7 Microbiota–epigenome interactions in neurodegeneration: insights from AD and PD

gut–brain axis represents an integrated systems-level regulatory network in which infection-driven dysbiosis, microbial metabolites, and inflammatory signaling converge to regulate miRNA expression, transcriptional programs, microglial activation, and synaptic function. Within this framework, gut microbiota act as upstream modulators of neuroimmune and epigenetic-like mechanisms that shape neurodegenerative vulnerability in AD and PD (**Table 3**).

**Table 3:** Microbiota–epigenome interactions in AD and PD.

Study type/Model	Treatment/Factor	Inflammatory pathway	Inflammatory markers	Epigenetic component	Key findings	Ref
Bioinformatics analysis of AD patient samples	Gut dysbiosis (NGS + transcriptomics)	MAPK, ErbB, Axon Guidance	Not specified	miRNAs: let-7c, miR-125b-2, miR-145	Dysbiosis correlates with altered miRNAs and AD pathways	(Asadifard et al., 2024)
Clinical (8 AD, 8 controls)	Natural gut microbiota	JNK, SNARE complex	MMSE scores	miRNAs: miR-3120-3p, miR-6529-5p, miR-124-3p, miR-323a-5p	Gut microbes and miRNAs linked to cognitive impairment	(Lin et al., 2025)
Rotenone-induced PD mouse model	Lactobacillus plantarum PS128	SOCS1–miR-155–NF-κB axis	IL-1β, TNF-α (inferred)	miR-155-5p, SOCS1	PS128 reduces inflammation via miR-155/SOCS1 modulation	(Lee et al., 2023)
Clinical + Rat PD model	Lactobacillus rhamnosus NCDC17	miR-146a–GDNF signaling	Neuroinflammation (GDNF levels)	miR-146a → GDNF	L. rhamnosus alters miR-146a and GDNF, affecting PD outcome	(Wei et al., 2021)

MAPK, mitogen-activated protein kinase; JNK, c-Jun N-terminal kinase; SNARE, soluble N-ethylmaleimide-sensitive factor attachment protein receptor; MMSE, Mini-Mental State Examination; IL-1β, interleukin-1 beta; TNF-α, tumor necrosis factor alpha; SOCS1–miR-155–NF-κB axis, suppressor of cytokine signaling 1–microRNA-155–nuclear factor kappa B signaling axis; GDNF, glial cell line-derived neurotrophic factor.

### 7.1 Microbiota–miRNA regulatory axis in neurodegeneration

Gut dysbiosis is strongly associated with altered miRNA expression that regulates synaptic signaling and neuroinflammation. Asadifard et al. (2024) reported reduced abundance of SCFA-producing bacteria (Firmicutes, Clostridia, Faecalibacterium) together with disruption of 619 shared gut–brain genes. These molecular changes converge on axon guidance, MAPK, and ErbB signaling pathways, which are essential for synaptic plasticity and neuronal stress responses. Key miRNAs such as let-7c, miR-125b-2, and miR-145 were identified as central regulatory nodes controlling these pathways (Asadifard et al., 2024). Lin et al. (2025) further demonstrated that circulating exosomal miRNAs (miR-124-3p, miR-6529-5p, miR-3120-3p, miR-323a-5p) correlate with microbial composition and cognitive decline. Importantly, these miRNAs regulate neurotrophin signaling, synaptic transmission, and cellular transport, indicating that gut microbes influence brain function through systemic miRNA-mediated regulatory circuits rather than isolated molecular changes.

### 7.2 Microbial regulation of miRNAs and neuroinflammation in PD

In PD, microbiota–miRNA interactions regulate neuroinflammatory signaling and dopaminergic neuronal survival. Zhang Lee et al. (2023) demonstrated that *Lactobacillus plantarum* PS128 reduces miR-155-5p expression, restoring SOCS1 levels and suppressing NF-κB/STAT1-mediated inflammation. This establishes a direct microbial–miRNA–transcription factor axis linking gut microbiota to CNS immune regulation. Wei et al. (2021) reported that miR-146a negatively regulates glial cell line-derived neurotrophic factor (GDNF), thereby reducing dopaminergic neuron survival. Elevated miR-146a levels were associated with gut dysbiosis, suggesting microbial control of neurotrophic signaling via post-transcriptional regulation. Zhao et al. (2021) further demonstrated that microbial toxins such as LPS activate TLR4–NF-κB signaling, sustaining inflammatory gene expression and promoting epigenetic-like transcriptional reprogramming.

### 7.3 Microbiota-driven transcriptomic reprogramming and synaptic plasticity

Microbial interventions induce widespread transcriptomic remodeling in brain and gut tissues, affecting synaptic plasticity, inflammation, and neuronal survival. Webberley et al. (2022) showed that a multi-strain probiotic mixture known as Lab4b, consisting of *Lactobacillus salivarius* CUL61, *Lactobacillus paracasei* CUL08, *Bifidobacterium bifidum* CUL20, and *Bifidobacterium animalis* subsp. lactis CUL34 upregulated synaptic genes including CPLX2, GRIA1, and NSMF, with a trend toward increased BDNF expression. These findings indicate that probiotics enhance synaptic plasticity primarily through transcriptional activation of neuronal connectivity pathways rather than amyloid clearance alone (**Table 4**). Webberley et al. (2023) further demonstrated that Lab4P reduced inflammatory gene expression while preserving hippocampal spine density under metabolic stress conditions. Hu and Shao (2022) showed that *Lactobacillus pentosus* upregulates BIRC3, which suppresses NLRC4 inflammasome activation and reduces IL-1β/IL-18-mediated pyroptosis. This highlights probiotic control of inflammasome-driven transcriptional programs involved in neuroinflammation. Chen et al. (2017) demonstrated that fructooligosaccharides reshape gut microbiota composition and regulate genes involved in neurotransmitter synthesis, mitochondrial function, and inflammatory signaling. These transcriptomic alterations were associated with reduced Aβ deposition and tau pathology, linking microbial metabolism to host gene regulation across the gut–brain axis. He et al. (2024) reported that Danggui Shaoyao San significantly suppresses IL-1β, TNF-α, and IL-6 via NF-κB inhibition while modulating gut microbial composition, particularly enriching Ligilactobacillus species (**Table 5**). Wang et al. (2022) demonstrated that Lactobacillus plantarum MA2 inhibits the TLR4/MYD88/NLRP3 signaling axis, resulting in reduced microglial activation, improved intestinal barrier integrity, and decreased amyloid pathology.

**Table 4:** Transcriptomic shifts and neuroinflammation via probiotics.

Study type/model	Treatment/intervention	Targeted pathway(s)	Inflammatory markers	Epigenetic involvement	Key results	Ref
Animal model (3xTg-AD mice) + HFD	Lab4b probiotic consortium	Neurogenesis, anti-inflammatory pathways	Systemic and local inflammatory responses	Not directly studied (but gene expression modulated)	Improved cognition and spine density; reduced inflammation; altered gut microbiota	(Webberley et al., 2022)
Animal model (3xTg-AD mice); in vitro (SH-SY5Y)	Lab4P probiotic consortium	Amyloid protection, inflammation, neuroplasticity	Anti-inflammatory gene expression (brain, in vitro)	Not specified, but gene expression changes in hippocampus	Improved cognition; protective against β-Amyloid toxicity; enhanced mRNA of synaptic genes	(Webberley et al., 2023)
In vitro cell model (SH-SY5Y neurons)	Lactobacillus pentosus S-PT84	BIRC3/NLRC4 pyroptosis signaling	IL-1β, IL-18, cleaved caspase-1, GSDMD-N	BIRC3 gene regulation (post-transcriptional, epigenetic relevance)	Reduced pyroptosis; restored BIRC3; suppressed inflammation via NLRC4 inhibition	(Hu and Shao, 2022)
Animal model (D-galactose + Aβ1–42 in rats)	Fructooligosaccharides from Morinda officinalis (OMO)	Microbiota-gut-brain axis; neurotransmitter synthesis; intestinal transcriptome	Cytokines in brain; inflammation-related genes in intestine	Transcriptomic shifts in gut and brain; altered gene expression	Improved memory and brain histology; reduced oxidative stress and inflammation; shifted microbiota; gene expression changes in gut and brain	(Chen et al., 2017)

HFD, high-fat diet; IL-1β, interleukin-1 beta; IL-18, interleukin-18; GSDMD-N, N-terminal fragment of gasdermin D; 3xTg-AD mice, triple-transgenic Alzheimer’s disease mouse model (APP, PSEN1, and tau mutations).

**Table 5:** Gut microbiota and epigenetic regulation of inflammatory pathways.

Study type and model	Treatment/intervention	Targeted pathway(s)	Inflammatory markers involved	Epigenetic involvement	Key results	Ref
Commentary, AD patients and molecular evidence	Gut-derived neurotoxins (e.g., LPS, bacterial amyloids, sncRNA)	Innate immunity, neurovascular signaling	Not explicitly named; general neuroinflammation	sncRNAs	Neurotoxins like LPS and sncRNAs cross barriers, enter CNS, and contribute to AD-related inflammation and degeneration.	(Zhao et al., 2021)
Experimental, d-galactose/AlCl3-induced AD rats	Probiotic (Lactobacillus plantarum MA2)	TLR4/MYD88/NLRP3 signaling	Not detailed in abstract	Not mentioned	MA2 improved cognition, reduced Aβ and inflammation, and reshaped microbiota.	(Wang et al., 2022)
Experimental, STZ-induced AD rats	Herbal compound (Danggui Shaoyao San)	Gut–brain metabolite modulation, Purine and Nicotinate metabolism	IL-1β, IL-6, TNF-α	Not mentioned	DSS enhanced memory, reduced inflammation and oxidative stress, and shifted microbiota and metabolites.	(He et al., 2024)

sncRNA, small non-coding RNA; IL-1β, interleukin-1 beta; IL-6, interleukin-6; TNF-α, tumor necrosis factor alpha; STZ-induced AD rats, streptozotocin-induced Alzheimer’s disease rat model; TLR4/MYD88/NLRP3 signaling, Toll-like receptor 4/myeloid differentiation primary response protein 88/NOD-like receptor family pyrin domain containing 3 signaling pathway.

### 7.4 Integrated inflammatory signaling and epigenetic-like regulation

Inflammatory signaling pathways act as central hubs connecting microbial dysbiosis to transcriptional and epigenetic regulation. PS128-mediated downregulation of miR-155-5p restores SOCS1 expression and suppresses NF-κB signaling (Lee et al., 2023), while BIRC3 activation inhibits NLRC4 inflammasome-mediated pyroptosis (Hu and Shao, 2022). This cascade can be summarized as:

gut dysbiosis → microbial metabolites/toxins → miRNA dysregulation → transcription factor activation (SOCS1, BIRC3, NF-κB, TLR4) → microglial activation → synaptic dysfunction

Zhao et al. (2021) demonstrated that persistent LPS exposure sustains NF-κB-driven inflammatory gene expression and induces epigenetic-like chromatin remodeling. Although not a classical epigenetic enzyme, NF-κB signaling can influence histone acetylation and chromatin accessibility.

Importantly, previous mechanistic frameworks also emphasize that chronic neuroinflammation is tightly linked with persistent activation of innate immune signaling cascades and downstream transcriptional reprogramming events, which collectively bridge microbial signals to long-term changes in brain immune tone (Zhao et al., 2021; Wang et al., 2025). Within this context, inflammatory mediators such as cytokines and pattern recognition receptor pathways act as functional intermediates between microbial stimuli and epigenetic-like chromatin regulation, reinforcing the concept that gut-derived signals can induce durable transcriptional memory in microglia and other CNS-resident cells (Zhao et al., 2021; Wang et al., 2025). He et al. (2024) and Wang et al. (2022) further confirm that suppression of NF-κB/TLR4/NLRP3 axes reduces neuroinflammation and restores microbial–metabolic balance.

### 7.5 Therapeutic implications

Microbiota-targeted therapies, including probiotics, prebiotics, and dietary interventions, represent promising strategies for NDDs. These interventions exert multi-level effects across miRNA regulation, transcriptional reprogramming, metabolic signaling, and neuroinflammatory suppression. Wei et al. (2021) highlight that excessive miR-146a signaling may worsen neurodegeneration by suppressing GDNF, emphasizing the importance of strain-specific probiotic selection. Zhang Lee et al. (2023) further demonstrate that PS128 restores dopaminergic signaling through miRNA modulation. Webberley et al. (2023) and Hu and Shao (2022) show that probiotic-derived metabolites directly influence neuronal gene expression and synaptic stability. Overall, this section supports a unified mechanistic framework in which gut microbiota regulate neurodegeneration through coordinated modulation of metabolites, miRNAs, transcriptional networks, inflammatory signaling, and synaptic integrity across AD and PD.

## 8 Infection-driven gut dysbiosis and barrier dysfunction as upstream determinants of systemic endotoxemia

Mounting clinical and experimental evidence suggests that systemic infections are consistently accompanied by measurable alterations in gut microbial ecology and intestinal barrier integrity. These changes extend beyond local gastrointestinal disturbances and are increasingly recognized as part of a reproducible host–microbiome response to infection. A synthesis of key clinical studies examining infection-associated dysbiosis, barrier dysfunction, and endotoxemia is summarized in **Table 6**.

**Table 6:** Infection-associated dysbiosis, barrier dysfunction, and microbial translocation signatures.

Infection context	Sample type	Dysbiosis signature (Composition)	Barrier dysfunction marker	Microbial translocation marker	Functional/clinical outcome	Ref
COVID-19 (sepsis)	Serum	Microbiota-linked metabolic shift (↑TMAO)	—	↑LPS	Septic shock severity	(Polat et al., 2024)
COVID-19 pneumonia	Serum	—	↑ZO-1	↑Endotoxin	Systemic inflammation	(Assimakopoulos et al., 2021)
COVID-19	Plasma	↑F/B ratio, ↓Bacteroides	↑FABP2	↑LPS, PGN	Gut barrier disruption	(Prasad et al., 2022)
COVID-19	Stool	↓Faecalibacterium, ↓Bifidobacteria	—	—	Disease severity linkage	(Yeoh et al., 2021)
Sepsis	Plasma	—	↑Zonulin	—	Increased intestinal permeability	(Klaus et al., 2013)
COVID-19	Plasma	Enterobacteriaceae enrichment	↑Zonulin	—	Cytokine elevation	(Souza et al., 2025)
AVL/HIV coinfection	Plasma	—	↑IFABP	↑LPS, sCD14	Immune activation	(Santos-Oliveira et al., 2013)
HIV therapy (MPI)	Plasma	—	—	↑LBP, sCD14	Monocyte activation	(Torres et al., 2014)
HIV therapy (mtDRV)	Plasma	—	—	↓LPS (suppressed VL)	Reduced T-cell activation	(BenMarzouk-Hidalgo et al., 2015)
HIV (perinatal)	Plasma	—	—	↑LPS, ↑LBP, ↑sCD14	Early immune activation	(Papasavvas et al., 2011)
HIV transmission (infants)	Plasma	—	↑Permeability	↑LPS	Infection acquisition risk	(Kourtis et al., 2013)
Dengue	Blood	↑Proteobacteria, ↑Escherichia	Leaky gut	↑LPS, ↑β-D-glucan	Severe disease progression	(Chancharoenthana et al., 2022)

COVID-19, coronavirus disease 2019; TMAO, trimethylamine N-oxide; LPS, lipopolysaccharide; ZO-1, zonula occludens-1; FABP2, fatty acid–binding protein 2 (intestinal fatty acid–binding protein); PGN, peptidoglycan; F/B ratio, Firmicutes-to-Bacteroidetes ratio; VL/HIV, visceral leishmaniasis/human immunodeficiency virus coinfection; LBP, lipopolysaccharide-binding protein; sCD14, soluble cluster of differentiation 14; β-D-glucan, beta-D-glucan.

### 8.1 Infection-associated dysbiosis: a reproducible compositional signature

Accumulating clinical and translational evidence suggests that infectious diseases can trigger a relatively conserved shift in gut microbial composition that may act as a biological signature of systemic immune imbalance. In patients with coronavirus disease 2019 (COVID-19), Yeoh et al. (2021) reported substantial alterations in intestinal microbiota compared with non-infected controls, regardless of therapeutic exposure. Specifically, microbial taxa known for their anti-inflammatory and immunoregulatory roles—such as *Faecalibacterium prausnitzii*, *Eubacterium rectale*, and members of the *Bifidobacterium genus*—were consistently reduced during active infection. Notably, these compositional disturbances did not immediately revert following viral clearance. Instead, depletion of these beneficial commensals persisted for several weeks after recovery, suggesting that infection-associated dysbiosis may outlast the acute phase of the disease. Furthermore, these microbiota shifts were linked with elevated systemic inflammatory markers, including C-reactive protein and liver-associated enzymes, indicating a close relationship between microbial imbalance and host immune responses (Yeoh et al., 2021). Parallel findings were documented by Prasad et al. (2022), who explored circulating microbial profiles in plasma samples obtained from hospitalized individuals with SARS-CoV-2 infection. Their analysis revealed systemic microbial imbalance characterized by a decline in Bacteroides populations alongside an increased Firmicutes-to-Bacteroidetes (F/B) ratio—a compositional feature frequently associated with inflammatory states and impaired epithelial integrity. In addition to taxonomic changes, infected patients exhibited significantly elevated levels of intestinal permeability markers such as fatty acid binding protein-2. Simultaneously, microbial-derived antigens, including LPS and peptidoglycan fragments, were detected at higher concentrations in the circulation. These findings strongly support the occurrence of microbial translocation from the intestinal lumen into the bloodstream during infection. The convergence of increased F/B ratio with heightened permeability markers suggests that compositional dysbiosis is functionally linked with epithelial barrier compromise, thereby facilitating systemic exposure to pro-inflammatory microbial products (Prasad et al., 2022). Further mechanistic insight into this reproducible microbial signature has been provided by Souza et al. (2025), who evaluated bacteriome dynamics in patients experiencing both acute and post-acute SARS-CoV-2 infection. Their study demonstrated marked alterations in microbial diversity that were accompanied by increased plasma concentrations of pro-inflammatory cytokines. Importantly, expansion of opportunistic bacterial groups, including Enterobacteriaceae and *Escherichia–Shigella*, showed a positive association with circulating zonulin levels. Zonulin is recognized as a key regulator of tight junction integrity and plays a central role in modulating epithelial permeability. The observed correlation suggests that infection-associated microbial changes may directly influence barrier stability by activating zonulin-mediated signaling pathways.

Consequently, dysbiosis-driven increases in zonulin may promote paracellular leakage, enabling the systemic dissemination of microbial metabolites and structural components that can further amplify immune activation through pattern recognition receptors such as TLR4 (Souza et al., 2025). Comparable infection-related dysbiotic patterns have also been observed in viral diseases beyond COVID-19. In patients with dengue infection, Chancharoenthana et al. (2022) identified evidence of gut-derived microbial translocation into the systemic circulation, particularly in individuals with severe clinical manifestations. Blood bacteriome profiling revealed an increased abundance of Proteobacteria and Escherichia species, accompanied by reduced representation of protective commensals such as Bifidobacterium. These taxonomic shifts occurred alongside elevated lactulose-to-mannitol ratios, indicating compromised intestinal barrier integrity, as well as increased circulating concentrations of LPS and β-D-glucan. Moreover, the presence of microbial components in plasma correlated with activation of innate immune cell populations, including natural killer cells and pro-inflammatory monocyte subsets (Chancharoenthana et al., 2022).

### 8.2 Barrier dysfunction and endotoxemia: functional signatures of infection-associated dysbiosis

While infection-associated dysbiosis is initially reflected by compositional shifts in the gut microbial community, its downstream impact becomes more evident through functional alterations that compromise intestinal barrier integrity. A disrupted epithelial interface allows microbial-derived molecules to escape the gut lumen and enter the systemic circulation, where they can modulate host immune responses. In this regard, clinical observations from COVID-19–associated pneumonia have provided early insight into the physiological consequences of gut barrier impairment. Assimakopoulos et al. (2021) reported significantly elevated circulating levels of both endotoxin and zonula occludens-1 (ZO-1) in hospitalized patients compared with healthy individuals. As ZO-1 is a key structural component of epithelial tight junctions, its presence in peripheral blood likely reflects damage to the intestinal epithelial layer and loosening of paracellular connections (Assimakopoulos et al., 2021). A similar disruption of mucosal integrity has also been documented in patients with sepsis. Klaus et al. (2013) demonstrated that circulating levels of zonulin—a physiological regulator of tight junction permeability—were significantly elevated in septic individuals relative to both postoperative controls and healthy subjects. Increased zonulin activity is associated with junctional disassembly and enhanced intestinal permeability, which may allow luminal microbial components to reach the bloodstream (Klaus et al., 2013). Further support for this mechanism comes from studies investigating co-infected populations. In patients with visceral leishmaniasis and human immunodeficiency virus (HIV), Santos-Oliveira et al. (2013) identified markedly increased plasma concentrations of LPS alongside elevated levels of I-FABP, a biomarker of enterocyte injury. Elevated I-FABP indicates epithelial cell damage within the gut mucosa, which may facilitate the movement of microbial endotoxins across the compromised barrier. Notably, circulating LPS levels in these patients were positively correlated with immune activation markers and inflammatory cytokines such as IL-6 and IL-8 (Santos-Oliveira et al., 2013).

Evidence derived from pediatric HIV cohorts provides additional clinical relevance to these observations. Papasavvas et al. (2011) reported increased plasma concentrations of LPS as well as host endotoxin-binding molecules, including soluble CD14 (sCD14) and LPS-binding protein (LBP), in perinatally infected infants during early life. Infants who experienced delayed initiation of antiretroviral therapy exhibited consistently higher levels of these microbial translocation markers than those treated earlier (Papasavvas et al., 2011). Comparable findings were reported in the Breastfeeding, Antiretrovirals, and Nutrition study by Kourtis et al. (2013), in which plasma LPS levels in infants were associated with an increased risk of HIV acquisition through breast-feeding. Elevated endotoxin concentrations prior to infection were also linked to reduced growth parameters (Kourtis et al., 2013). The influence of therapeutic regimens on microbial translocation markers has also been explored in adult HIV populations. Torres et al. (2014) compared patients receiving protease inhibitor monotherapy with those undergoing combination antiretroviral therapy and observed higher levels of microbial translocation markers, including LBP, in the monotherapy group. These alterations were accompanied by increased concentrations of soluble monocyte activation markers such as sCD14 and elevated IL-6 levels (Torres et al., 2014). Conversely, BenMarzouk-Hidalgo et al. (2015) examined HIV-infected individuals undergoing ritonavir-boosted darunavir monotherapy over a 24-month follow-up period and found that reductions in circulating LPS concentrations were mainly observed in patients maintaining continuous viral suppression (BenMarzouk-Hidalgo et al., 2015). Additional clinical data linking endotoxemia to disease severity have been reported in patients with COVID-19–related septic shock. Polat et al. (2024) demonstrated that circulating LPS levels were significantly higher in septic patients compared with non-septic infected individuals and healthy controls. Elevated endotoxin concentrations were accompanied by increased levels of trimethylamine N-oxide (TMAO), a microbiota-derived metabolite associated with inflammatory states. The concurrent rise in LPS and TMAO in severely ill patients suggests that gut-derived microbial products may contribute to systemic inflammatory burden and clinical deterioration during infection (Polat et al., 2024).

## 9 Microbiota-derived metabolites as epigenetic regulators of host immune programming

Beyond compositional shifts in microbial communities and barrier dysfunction, accumulating evidence suggests that microbiota-derived metabolites actively participate in reshaping host immune responses at the epigenetic level. In particular, SCFAs such as butyrate have been shown to modulate HDAC activity, thereby altering chromatin accessibility and inflammatory gene transcription (Chang et al., 2014; Parada‐Venegas et al., 2025). Similarly, bacterial LPS can initiate TLR4-dependent signaling cascades that induce histone methylation and acetylation changes at the promoters of pro-inflammatory genes, enabling sustained transcriptional responsiveness (Yoshida et al., 2015). In parallel, microbiota-derived tryptophan metabolites have emerged as endogenous ligands for the aryl hydrocarbon receptor (AhR), influencing epithelial integrity and immune homeostasis through transcription factor-mediated gene regulation (Scott et al., 2020; Tan et al., 2022).

### 9.1 Butyrate-mediated HDAC inhibition and LPS-driven chromatin reprogramming in host immune cells

A growing body of mechanistic evidence indicates that microbiota-derived SCFAs, particularly butyrate, function not only as metabolic substrates but also as epigenetic regulators capable of modifying host inflammatory responses through histone-modifying enzymes. One of the earliest demonstrations of this concept was provided by Chang et al. (2014), who showed that butyrate suppresses LPS-induced inflammatory mediator production in intestinal macrophages through inhibition of HDAC activity, thereby promoting immune tolerance toward commensal microbiota without broadly impairing immune competence (Chang et al., 2014). Consistently, Jiang et al. (2020) reported that sodium butyrate attenuates LPS-induced inflammatory gene expression in macrophages by suppressing NF-κB activation and NLRP3 inflammasome signaling, while simultaneously preventing histone deacetylation at inflammatory gene loci (Jiang et al., 2020). Parallel observations in epithelial systems have further indicated that SCFAs such as sodium butyrate and sodium propionate can selectively inhibit class I HDAC isoforms and enhance histone H3 acetylation at regulatory regions involved in inflammatory signaling pathways (Silva et al., 2018). Beyond immune cells, transcriptional analyses have revealed that butyrate induces widespread gene expression changes in metabolically active tissues, including endothelial and skeletal muscle cells, consistent with its activity as a potent HDAC inhibitor (Chriett et al., 2019). These chromatin-level changes appear to extend to pathological contexts. For instance, Zhao et al. (2025) demonstrated that butyrate-mediated suppression of HDAC2 expression attenuates IL-6/STAT3 signaling in hepatic stellate cells, thereby reducing fibrotic activation in biliary atresia models. Similarly, recent transcriptomic data suggest that butyrate treatment can modify histone lactylation patterns associated with inflammatory gene expression in pulmonary immune cells, leading to reduced MAPK- and Janus kinase–signal transducer and activator of transcription-mediated inflammatory signaling in experimental models of chronic obstructive pulmonary disease (Jiang et al., 2025). Comparable epigenetic modulation has also been reported in inflamed intestinal tissues, where butyrate targets HDAC3 activity in monocytes and macrophages, resulting in increased H3K9 acetylation and reduced secretion of TNF-α and IL-6 in ex *vivo* inflammatory bowel disease biopsies (Parada‐Venegas et al., 2025). At the endocrine level, HDAC inhibition by butyrate has been shown to restore histone H3 and H4 acetylation in pancreatic islets exposed to chronic IL-1β stimulation, thereby preserving insulin secretory function and attenuating inflammation-associated transcriptional dysregulation (Pedersen et al., 2024). In parallel, pathogen-associated molecular patterns such as LPS have been shown to induce persistent epigenetic changes through chromatin remodeling pathways. Experimental studies have demonstrated that LPS stimulation enhances histone methylation marks, particularly H3K4me2, at promoters of pro-inflammatory cytokine genes including IL-6 and TNF-α, facilitating their transcriptional activation (Zhao et al., 2019). Additionally, LPS exposure has been reported to dynamically regulate HDAC expression in macrophages, thereby modulating the transcriptional amplitude of inflammatory mediators such as COX-2 and Ccl2 at the promoter level (Aung et al., 2006). Indeed, Yoshida et al. (2015) identified a role for the transcription factor ATF7 in mediating LPS-induced epigenetic memory in macrophages through regulation of repressive histone H3K9me2 marks, leading to sustained basal expression of innate immune genes following initial pathogen exposure.

### 9.2 Tryptophan-derived microbial metabolites as AhR-mediated epigenetic modulators

In addition to SCFAs, gut microbiota generates a wide range of small-molecule metabolites from dietary amino acids that may participate in host transcriptional regulation. Among these, tryptophan-derived microbial catabolites have increasingly been suggested to act as endogenous modulators of the AhR, a ligand-activated transcription factor involved in immune homeostasis and epithelial barrier maintenance. Emerging experimental evidence indicates that microbiota-derived indole compounds can activate AhR-dependent signaling cascades, thereby influencing downstream gene expression programs related to oxidative stress responses, inflammation, and mucosal integrity (Tan et al., 2022). Several mechanistic studies have demonstrated that microbial tryptophan metabolites, including indole-3-aldehyde, indole-3-ethanol, and indole-3-pyruvate, may exert protective effects on epithelial barrier function through AhR activation in experimental models of intestinal inflammation (Scott et al., 2020). Activation of AhR by these metabolites has been associated with stabilization of apical junctional complexes and actin-associated cytoskeletal regulators, suggesting a potential role in maintaining intestinal permeability under inflammatory conditions. Although the precise molecular targets of these metabolites remain incompletely characterized, available data indicate that AhR-dependent transcriptional responses may contribute to improved barrier resilience by regulating genes involved in epithelial integrity and immune tolerance.

Importantly, not all microbiota-derived metabolites interact with AhR in an equivalent manner. Detailed *in vitro* characterization of gut microbial tryptophan catabolites has shown that these molecules exhibit varying agonistic or antagonistic activities toward AhR, resulting in ligand-specific transcriptional outcomes (Vyhlídalová et al., 2020). Reporter gene assays and ligand-binding analyses have demonstrated that several indole derivatives can induce nuclear translocation of AhR and promote heterodimer formation with the AhR nuclear translocator, ultimately enhancing binding to target gene promoters such as CYP1A1. Interestingly, recent experimental work indicates that butyrate may further modulate AhR-dependent transcriptional responses through chromatin-level mechanisms. While butyrate does not directly function as an AhR ligand, it appears capable of enhancing receptor activity in the presence of endogenous tryptophan-derived agonists by inhibiting HDAC at AhR target gene promoters (Modoux et al., 2022). Chromatin immunoprecipitation analyses in human intestinal explants have suggested that HDAC inhibition by butyrate may facilitate increased recruitment of AhR to responsive genomic regions, thereby amplifying transcriptional activation mediated by microbial indole compounds.

## 10 Peripheral immune imprinting and epigenetic reprogramming of microglia across the lifespan

Peripheral immune signals originating from infection, dysbiosis, or systemic inflammation are increasingly recognized as modulators of CNS immune homeostasis (Mou et al., 2022). In this context, microglia have gained attention as long-lived innate immune cells capable of integrating prior environmental exposures into altered functional phenotypes over extended temporal scales. This adaptive capacity, often conceptualized as innate immune memory, is thought to arise not from permanent genetic alteration but from dynamic reconfiguration of chromatin architecture that regulates stimulus-dependent transcriptional programs (Tay et al., 2017; Haley et al., 2019). A summary of representative experimental studies investigating epigenetic mechanisms underlying microglial immune memory and inflammatory priming is provided in **Table 7**. At the mechanistic level, epigenetic restructuring of enhancer regions has been proposed as a central process through which innate immune cells acquire heightened or attenuated responsiveness following inflammatory exposure.

**Table 7:** Experimental and mechanistic evidence supporting peripheral immune imprinting and epigenetic reprogramming of microglia across the lifespan.

Experimental model	Immune trigger/stimulus	Epigenetic or regulatory mechanism	Neuroimmune/biological outcome	Conceptual implication	Ref
Primary microglia, PD mouse model, postmortem human PD brain	LPS priming followed by manganese (Mn) stress	Enhanced deposition of H3K27ac, H3K4me3, H3K4me1	Exaggerated inflammatory cytokine responses in primed microglia	Epigenetic reprogramming mediates microglial immune memory	(Huang et al., 2023)
Macrophage and B-cell lineage systems	Lineage transcription factor cooperation	PU.1-driven establishment of H3K4me1-marked enhancer regions	Specification of cell-type gene regulatory programs	Lineage-determining TF networks prime immune enhancer landscapes	(Heinz et al., 2010)
Embryonic stem cells and adult mouse tissues	Developmental transcriptional programs	H3K27ac distinguishes active vs poised enhancers	Prediction of enhancer activity and cellular state	Histone acetylation marks regulate enhancer activation states	(Creyghton et al., 2010)
Dendritic cells analyzed by high−throughput ChIP	Pathogen stimulation	Dynamic transcription factor binding prior to gene activation	Pre-bound regulatory complexes enable rapid gene induction	Immune genes may be primed epigenetically before stimulation	(Garber et al., 2012)
Activated macrophages	TLR4 signaling	De novo enhancer formation with H3K4 methylation and enhancer transcription	Activation of inflammatory gene expression programs	Enhancer remodeling coordinates macrophage immune activation	(Kaikkonen et al., 2013)
Differentiated macrophages	Environmental immune stimulation	Formation of latent enhancers acquiring TF binding and histone marks	Enhanced transcriptional response upon restimulation	Latent enhancer activation creates epigenomic memory	(Ostuni et al., 2013)
Macrophage inflammatory gene networks	Endotoxin stimulation	PU.1−dependent enhancer activity with p300 acetyltransferase recruitment	Regulation of inflammatory transcription programs	Combinatorial transcription factor networks regulate immune enhancers	(Ghisletti et al., 2010)
Mouse microglia analyzed by ATAC−seq, ChIP−seq and RNA−seq	Repeated LPS exposure or genotoxic stress	Gain or loss of permissive enhancer-associated chromatin marks	Primed or tolerized microglial phenotypes	Microglial innate immune memory linked to epigenetic enhancer remodeling	(Zhang et al., 2022)
In vivo cortical microglia imaging	Blood–brain barrier disruption or injury	Activity-dependent microglial activation	Rapid microglial surveillance and response to tissue damage	Microglia function as dynamic environmental immune sensors	(Nimmerjahn et al., 2005)
Neonatal rat infection model	Early bacterial infection followed by adult LPS challenge	Persistent neuroimmune sensitization	Impaired hippocampal-dependent contextual memory	Early immune exposure predisposes to later cognitive vulnerability	(Bilbo et al., 2006)
Fetal sheep microglia model	Prenatal LPS exposure followed by secondary stimulation	Altered transcriptomic and HDAC-related pathways	Amplified cytokine responses in primed microglia	Prenatal inflammation programs long-term microglial responses	(Cao et al., 2015)
Young vs aged mice	Peripheral LPS challenge	Age-associated amplification of inflammatory signaling	Prolonged sickness and depressive-like behavior	Aging enhances brain sensitivity to peripheral immune activation	(Godbout et al., 2008)
Isolated microglia from aged mouse brain	Peripheral endotoxin exposure	Increased cytokine induction and immune receptor expression	Hyper-responsive microglial inflammatory phenotype	Age-associated microglial priming contributes to neuroinflammation	(Henry et al., 2009)
Mouse developmental inflammation model	Neonatal LPS exposure followed by adult challenge	Long-term neuroimmune and HPA-axis alterations	Impaired spatial memory and altered emotional behavior	Early-life inflammation induces lifelong neuroimmune vulnerability	(Dinel et al., 2014)
Mouse model of systemic immune activation (PolyI:C)	Prenatal and adult immune challenge	Chronic inflammatory signaling and APP dysregulation	Alzheimer-like neuropathology during aging	Systemic immune activation may initiate neurodegenerative pathology	(Krstic et al., 2012)

PD, Parkinson’s disease; TLR4, Toll-like receptor 4; LPS, lipopolysaccharide; HDAC, histone deacetylase; HPA axis, hypothalamic–pituitary–adrenal axis; ATAC-seq, assay for transposase-accessible chromatin sequencing; ChIP-seq, chromatin immunoprecipitation sequencing; RNA-seq, RNA sequencing.

In myeloid lineage cells, lineage-determining transcription factors such as PU.1 cooperate with signal-responsive transcription factors to establish accessible chromatin domains at distal regulatory loci marked by histone H3 lysine 4 monomethylation (H3K4me1), thereby facilitating nucleosome remodeling and priming inflammatory gene enhancers without necessarily initiating immediate transcriptional output (Ghisletti et al., 2010; Heinz et al., 2010). Consistent with this hierarchical framework, genome-scale chromatin immunoprecipitation studies have demonstrated that a substantial proportion of transcription factor–DNA interactions associated with inflammatory gene programs are already established prior to pathogen exposure (Garber et al., 2012). Such pre-bound complexes, frequently localized at immediate-early response elements, may act as transcriptional placeholders that enable rapid gene activation upon secondary environmental stimulation. Inflammatory engagement of pattern-recognition receptors, including Toll-like receptor 4 through bacterial LPS, has been shown to further remodel this enhancer landscape. Activation of macrophages is associated with the emergence of newly primed regulatory regions characterized by deposition of mono- and dimethylated H3K4 marks, processes that appear to involve histone methyltransferases of the MLL family (Kaikkonen et al., 2013). In certain contexts, transcription of enhancer-associated RNA precedes detectable histone methylation at these loci, suggesting that chromatin priming may be initiated prior to overt transcriptional changes in adjacent genes. Beyond inducible enhancer formation, differentiated innate immune cells may harbor previously unmarked genomic regions termed latent enhancers that acquire transcription factor occupancy and active histone modifications only following environmental stimulation (Ostuni et al., 2013).

Enhancer activity states may be further distinguished by histone acetylation marks such as H3K27ac, which are generally associated with transcriptionally engaged regulatory elements and have recently been implicated in the establishment of inflammatory memory and epigenetic priming in microglial cells following immune stimulation (Huang et al., 2023). Regions marked solely by H3K4me1 are often considered inactive or poised, whereas co-enrichment with H3K27ac reflects active participation in gene transcription programs (Creyghton et al., 2010). While many of these observations have been derived from peripheral macrophage models, emerging data suggest that comparable chromatin-level mechanisms may operate within microglia following systemic immune exposure. Microglial cells, as long-lived tissue-resident macrophages of the CNS, continuously survey their microenvironment even under homeostatic conditions through highly motile processes capable of rapidly responding to perturbations (Nimmerjahn et al., 2005). Experimental studies indicate that systemic inflammatory events occurring during sensitive developmental windows can exert lasting effects on microglial reactivity that remain functionally silent under basal conditions.

Neonatal exposure to peripheral bacterial infection has been associated with hippocampal-dependent memory impairments that become apparent only after a secondary immune challenge in adulthood (Bilbo et al., 2006). Similarly, postnatal inflammatory stimulation has been linked to alterations in emotional behaviour and impaired spatial memory following re-exposure to LPS later in life, despite minimal detectable deficits after the initial insult (Dinel et al., 2014). These findings suggest that early-life peripheral immune activation may induce a latent susceptibility state rather than immediate neuropathology. At the cellular level, this latent vulnerability appears to involve persistent transcriptional and epigenetic alterations in microglia following systemic inflammatory exposure. Models of fetal neuroinflammation have demonstrated that microglia isolated from *in vivo* LPS-exposed animals exhibit amplified cytokine responses upon subsequent *in vitro* stimulation, accompanied by differential regulation of inflammatory and metabolic pathways (Cao et al., 2015). Alterations in HDAC isoforms observed after repeated immune challenge further support the possibility that inflammatory memory in microglia may involve chromatin-level reprogramming rather than transient transcriptional activation alone (Cao et al., 2015). In agreement with this interpretation, genome-wide epigenomic profiling has shown that inflammatory priming is associated with enrichment of permissive epigenetic marks at enhancer regions, whereas tolerized responses may involve loss of such marks, collectively giving rise to distinct transcription factor networks and microglial phenotypes (Zhang et al., 2022). The functional consequences of such priming may become increasingly evident with advancing age. Aging has been associated with exaggerated neuroinflammatory responses to peripheral immune stimulation, including prolonged sickness behaviour and heightened induction of inflammatory mediators following systemic LPS administration (Godbout et al., 2008). Peripheral LPS challenge has also been shown to induce greater expression of cytokines such as interleukin-1β and interleukin-10, as well as increased major histocompatibility complex class II expression in microglia isolated from aged brains compared to younger counterparts (Henry et al., 2009). Beyond altered immune responsiveness, systemic immune challenges during sensitive developmental periods have been implicated in the initiation or acceleration of neuropathological processes later in life. Prenatal immune activation using viral mimetics has been shown to predispose mice to develop Alzheimer-like neuropathological features during aging, including increased amyloid precursor protein accumulation, altered tau phosphorylation, and sustained microglial activation (Krstic et al., 2012). When followed by a second immune challenge in adulthood, these alterations were further exacerbated, suggesting that repeated inflammatory events may interact to amplify neurodegenerative trajectories over time.

## 11 Conclusions and future perspectives

This review highlights growing evidence that infection-associated gut dysbiosis may contribute to long-term variability in neuroinflammatory susceptibility by influencing systemic immune homeostasis. A key mechanistic insight is that microbiota-derived metabolites, including SCFAs and tryptophan-derived indole compounds, may interact with host epigenetic machinery, thereby linking peripheral immune activity to sustained transcriptional regulation in innate immune cells. Microglia represent a central interface in this process due to their longevity and capacity for functional adaptation. Experimental studies suggest that early-life or repeated immune challenges may induce persistent changes in microglial responsiveness, potentially shaping later neuroinflammatory outcomes. However, current evidence remains largely derived from preclinical models and associative human studies, limiting causal interpretation. Future research should focus on longitudinal, multi-omics human studies integrating microbiome, immune, and brain readouts to establish causal relationships and identify modifiable neuroimmune pathways. Such approaches will be essential to determine whether microbiota-driven epigenetic and immune programming represents a targetable mechanism in NDDs.
